# Identification of m6A/m5C-related lncRNA signature for prediction of prognosis and immunotherapy efficacy in esophageal squamous cell carcinoma

**DOI:** 10.1038/s41598-024-58743-y

**Published:** 2024-04-08

**Authors:** Jianlin Wang, Huiwen Ren, Chao Xu, Bo Yu, Yiling Cai, Jian Wang, Xinye Ni

**Affiliations:** 1 Department of Radiotherapy, The Affiliated Changzhou Second People’s Hospital of Nanjing Medical University, Changzhou, 213003 Jiangsu China; 2 Department of Radiotherapy, Jiangyin People’s Hospital, Jiangyin, 214400 Jiangsu China; 3 Center for Medical Physics, Nanjing Medical University, Changzhou, 213003 Jiangsu China

**Keywords:** Esophageal squamous cell carcinoma, m6A, m5C, Prognosis, lncRNA, Biomaterials, Functional genomics, Genomics

## Abstract

N6-methyladenosine (m6A) and 5-methylcytosine (m5C) RNA modifications have garnered significant attention in the field of epigenetic research due to their close association with human cancers. This study we focus on elucidating the expression patterns of m6A/m5C-related long non-coding RNAs (lncRNAs) in esophageal squamous cell carcinoma (ESCC) and assessing their prognostic significance and therapeutic potential. Transcriptomic profiles of ESCC were derived from public resources. m6A/m5C-related lncRNAs were obtained from TCGA using Spearman’s correlations analysis. The m6A/m5C-lncRNAs prognostic signature was selected to construct a RiskScore model for survival prediction, and their correlation with the immune microenvironment and immunotherapy response was analyzed. A total of 606 m6A/m5C-lncRNAs were screened, and ESCC cases in the TCGA cohort were stratified into three clusters, which showed significantly distinct in various clinical features and immune landscapes. A RiskScore model comprising ten m6A/m5C-lncRNAs prognostic signature were constructed and displayed good independent prediction ability in validation datasets. Patients in the low-RiskScore group had a better prognosis, a higher abundance of immune cells (CD4 + T cell, CD4 + naive T cell, class-switched memory B cell, and Treg), and enhanced expression of most immune checkpoint genes. Importantly, patients with low-RiskScore were more cline benefit from immune checkpoint inhibitor treatment (*P* < 0.05). Our findings underscore the potential of RiskScore system comprising ten m6A/m5C-related lncRNAs as effective biomarkers for predicting survival outcomes, characterizing the immune landscape, and assessing response to immunotherapy in ESCC.

## Introduction

Esophageal carcinoma (EC) is a highly lethal gastrointestinal malignancy worldwide, which occurs an estimated 604,100 newly diagnosed cases and 544,100 individual deaths in 2020^1^. Esophageal squamous cell carcinoma (ESCC) represents the major histological subtype, constituting approximately 90% of all incident EC cases annually. The prevailing therapeutic approach for advanced-stage patients currently contains platin-based chemotherapy, either as a standalone regimen or in conjunction with checkpoint inhibitors. Notably, the integration of PD1 immunotherapy has yielded substantial clinical benefit, particularly within the subset of patients exhibiting tumor proportion scores (TPS) more than 1% or combined positive scores (CPS) higher than 10^2^. Nevertheless, despite breakthroughs in ESCC diagnosis and therapeutic intervention, the prognosis for advanced patients remains unsatisfactory owing to the clandestine onset of symptoms at an early stage and the propensity for metastatic dissemination. Thus, the emergence of a molecular phenotyping-based prognostic risk model offers useful information for the identification and differentiation of high-risk individuals, potentially leading to enhanced medical outcomes.

RNA modifications have been confirmed as a critical epigenetic mechanism for post-transcriptional gene regulation via affecting RNA splicing, stability, and translation. To date, over 170 different types of natural modification have been identified, and N6-methyladenosine (m6A) represents the most prevalent methylated alteration that occurs in all types of RNAs, including cording mRNA, tRNA, rRNA, and non-coding RNA. The process of m6A is determined by RNA methyltransferases (METTL3, METTL14), demethylases (FTO and ALKBH5), and recognized by methylation binding proteins (YTHDF1, YTHDC2, and IGF2BP1)^3^. Another type of RNA modification, 5-Methylcytosine (m5C) is also widespread in various RNAs but most abundant in eukaryotic tRNAs and rRNAs. It is mainly catalyzed by methyltransferase of DNMT2 and NSUN family, demethylated by methylcytosine dioxygenase TET2^4^. ALYREF recognizes m5C-methylated mRNA, and YBX1 interacts with m5C methylated site to regulate maternal mRNA stabilization. Deregulation of m6A and m5C regulators is reported in diverse human cancers and is intimately linked to oncogenic or tumor-suppressive functions, such as processes of proliferation, spreading, invasion, and immunity^5,6^. For example, METTL3 and METTL14 have been implicated as overexpressed and act as oncogenes in most cancer types. METTL3 could methylate BCL-2 and c-Myc mRNAs to increase their stability and expression, thus suppressing cancer cells apoptosis, and promoting disease progression^7^. m6A demethylase FTO displays the oncogenic function via reducing the tumor suppressor mRNA level, resulting in cancer cell differentiation and tumor growth^8^. The m6A regulators YTHDF1 and IGF2BP1 are overexpressed in ESCC. Increased expression of YTHDF1 and HNRNPC in ESCC could be utilized as a prognostic predictor^9^. IGF2BP1 interacted with cofactors RPS15 to promote core proteins translation of p38 MAPK signaling in an m6A-dependent manner^10^. In the case of m5C, the level of m5C is closely related to carcinogenesis, and NSUN2 could promote cancer cell proliferation via up-regulating the m5C levels. In ESCC patients, NSUN2 promotes cancer development and chemo-resistance via the mRNA-m5C modification of cancer-related genes and enhances their expression, such as TIGAR^11^ and GRB2^12^. Mechanically, elevated GRB2 levels increased the activation of downstream pathways PI3K and ERK in the LIN28B-dependent manner^12^. TIGAR activates the pentose phosphate pathway to generate more reductants, thus protecting cancer cells from ROS damage^11^.

Long non-coding RNAs (lncRNAs) are RNA molecules that do not encode proteins but exert a precise regulatory function via interacting with various target mRNAs, regulatory factors, and sponging microRNAs. The tissue-specific and condition-specific expression modes of lncRNAs implicated their potential use as biomarkers for early diagnosis and therapy outcome monitoring in cancer. m6A and m5C can also modify lncRNAs, thereby affecting tumor genesis and progression. In addition, risk models based on m6A or m5C-related lncRNAs were also developed for prognosis prediction. For example, Li et al. reported a prognostic risk model for low-grade glioma based on 8 m6A/m5C methylated lncRNAs signature that could predict survival and infiltration of immune cells^13^. By consensus clustering analysis of colorectal cancer samples, Song et al. established an m6A/m5C-related lncRNAs signature that displays accurate capability in predicting cancer prognosis, immune-stromal microenvironment, clinicopathological features, and immunotherapy efficacy^14^. Despite these findings, the advances in exploring the prevalence and molecular function of m6A/m5C methylated lncRNAs remain elusive.

In this study, we extracted transcriptome profiles from The Cancer Genome Atlas (TCGA) database, then conducted bioinformatics analysis to identify m6A/m5C -related lncRNAs signatures associated with ESCC prognosis. Moreover, we established the m6A/m5C lncRNAs-based RiskScore system for survival prediction and used the Gene Expression Omnibus (GEO) dataset for model validation. The association of RiskScore, immune microenvironment, and immune molecules were analyzed. Notably, we assess the predictive value of RiskScore in immunotherapy efficacy. Our findings highlight the potential role of m6A/m5C-related lncRNAs for clinical prognostic prediction in ESCC.

## Methods

### Data processing of transcriptomic profiles

RNA sequencing data associated with ESCC were derived from TCGA (https://xenabrowser.net/datapages/). Corresponding clinical information, encompassing overall survival times, age, and gender was also collected from TCGA. This dataset comprising 81 ESCC samples was set as a training set, as detailed in Table 1. Meanwhile, GSE53622^15^ dataset related to ESCC was obtained from GEO using the GEOquery package and was tested on the platform of Agilent-038314 CBC Homo sapiens lncRNA-mRNA microarray V2.0. This dataset consisted of 120 samples, of which contain clinical data.

**Table 1:** Clinical information of TCGA-ESCC and GSE53622 dataset.

		TCGA-ESCC	GSE53622
OS	Alive	65	54
	Dead	31	66
Age	≤ 60	61	60
	> 60	35	60
Gender	Male	81	96
	Female	15	24
Stage T	T1	7	8
	T2	34	14
	T3	50	96
	T4	5	2
Stage N	N0	52	58
	N1	29	40
	N2	5	18
	N3	–	4
	NX	10	–
Stage M	M0	85	–
	M1	5	–
	MX	5	–
Stage	I	7	8
	II	56	60
	III	27	52
	IV	5	–
Tobaco	Yes	–	69
	No	–	52
Alcohol	Yes	–	64
	No	–	56

### Identification of m6A/m5C-associated lncRNAs

We obtained m6A or m5C-related genes from previously published sources^16^. We classified the genes from transcriptome sequencing as either lncRNAs or mRNAs based on annotation information. To assess the expressed correlations between lncRNAs and m6A/m5C-related genes, we conducted Spearman's correlation analysis. We screened for m6A/m5C-lncRNA pairs with an absolute correlation coefficient greater than 0.3 and a p-value less than 0.05, selecting the corresponding lncRNAs as candidate molecules.

### LncRNA target genes prediction

LncRNAs modulate the target gene expression through co-localization and co-expression mechanisms. Here, we predicted the lncRNA target genes according to previous research^17,18^. We screened for protein-coding genes located within 10 kb of each lncRNA to explore their potential function. A correlation matrix was generated by computing the coefficient between all pairs of lncRNAs and mRNAs. We designated genes with an R^2^ > 0.95 as lncRNA target genes.

Furthermore, we utilized the online tools lncATLAS (http://lncatlas.crg.eu/)^19^ and lncSLdb^20^ (http://bioinformatics.xidian.edu.cn/lncSLdb/index.jsp) to determine the cellular localization of m6A/m5C-associated lncRNAs.

We performed gene ontology (GO) and Kyoto encyclopedia of genes and genomes (KEGG) analysis on these lncRNA target genes using the clusterProfile package, and the parameters were set as follows: pAdjust Method = 'BH', p-value Cutoff = 0.05, and q-value Cutoff = 0.2.

### Screening of prognosis-related lncRNAs

To classify ESCC samples from the TCGA dataset, we used the ConsensusClusterPlus package to generate a consistency matrix based on the expression of m6A/m5C-associated lncRNAs. Differential expression analysis of lncRNAs in the TCGA dataset was performed on log2-transformed TPM count data using the IOBR package^21^. Clustering consistency was evaluated using the k-means algorithm, with parameters including Euclidean distance, seed = 1100, and k.max = 10.

The survival package was employed for Cox analysis of m6A/m5C-associated lncRNAs in the TCGA-ESCC dataset, and a significance threshold of *P* < 0.05 was applied to identify prognostic genes.

### Construction of m6A/m5C-lncRNAs RiskScore model

Using the samples and corresponding genes in the TCGA-ESCC dataset, we conducted lasso analysis with the cv.glmnet function of the lars package. In the lasso analysis, parameters were set as “family = cox”, and genes with a coefficient value not equal to zero were retained. The RiskScore was defined as the sum of the product of the lasso score coefficient and gene expression, formulated as follows:

${\text{RiskScore}}= \sum_{i=1}^{n}expression \,\,of \,\,gene\,\, i \,\,* \,\,lasoo \,\,coefficient \,\,of \,\,gene\,\, i$ or, ${\text{RiskScore}}= \sum_{i=1}^{n}{exp}_{i}*{coef}_{i}$, where the *exp*_*i*_ represents the *ith* gene expression value (log2(TPM + 1)), and *coef*_*i*_ represents the lasso regression coefficient of the *ith* gene.

To validate the prediction accuracy of the RiskScore, we classified ESCC samples in the TCGA dataset into high- and low-RiskScore groups based on the median RiskScore. Kaplan–Meier curves were used to predict overall survival times between these groups, and receiver operating characteristic (ROC) curves were generated with corresponding AUC values calculated to assess predictive ability. The performance of the RiskScore model was also validated using the GSE53622 dataset.

### Immune landscape analysis

To evaluate the infiltration of immune cells and stromal cells in different clusters and subgroups, we calculated the ESTIMATE immune scores and stromal scores. Additionally, we utilized the ssGSEA, CIBERSORT, and xCell methods to assess the composition of the tumor microenvironment.

### Statistical analysis

#### Correlation analysis

The Peasron correlation test was performed using the R function corr.test. The P values of the association tests were corrected using FDR. In this paper, Pearson's correlation coefficient was used for testing. Pearson's correlation coefficient is a method used to measure the linear correlation between two variables. Its value is between -1 and 1. When the correlation coefficient is positive, it indicates that there is a positive correlation between the two variables. When the correlation coefficient is negative, it indicates that there is a negative correlation between the two variables. When the correlation coefficient is 0, it indicates that there is no linear correlation between the variables. (ns: *p* > 0.05, *p ≤ 0.05, **p ≤ 0.01,***p ≤ 0.001,****p ≤ 0.0001).

### Kaplan–Meier survival estimates

Kaplan–Meier survival estimation is a nonparametric method that estimates the probability of survival based on the observed survival time. Kaplan–Meier curves can be used to visualize survival differences across multiple categories, and the KM curve is a step function that reflects the cumulative probability of survival over time. The curve is horizontal during periods when no events occur and then decreases vertically as the survival function changes with each event.

In conclusion, Spearman's correlation was computed using the corr.test in R, with P values corrected using FDR. Wilcoxon tests were employed for pairwise comparisons. Kaplan–Meier curves were generated for prognosis analysis, with the log-rank test determining significant differences. Multivariate Cox regression analysis was conducted to identify independent predictive factors, including RiskScore, age, gender, and pathology stage. All statistical analyses were two-sided, and a significance level of *P* < 0.05 was applied.

## Results

### Screening m6A/m5C-related lncRNAs

**Figure 1:**
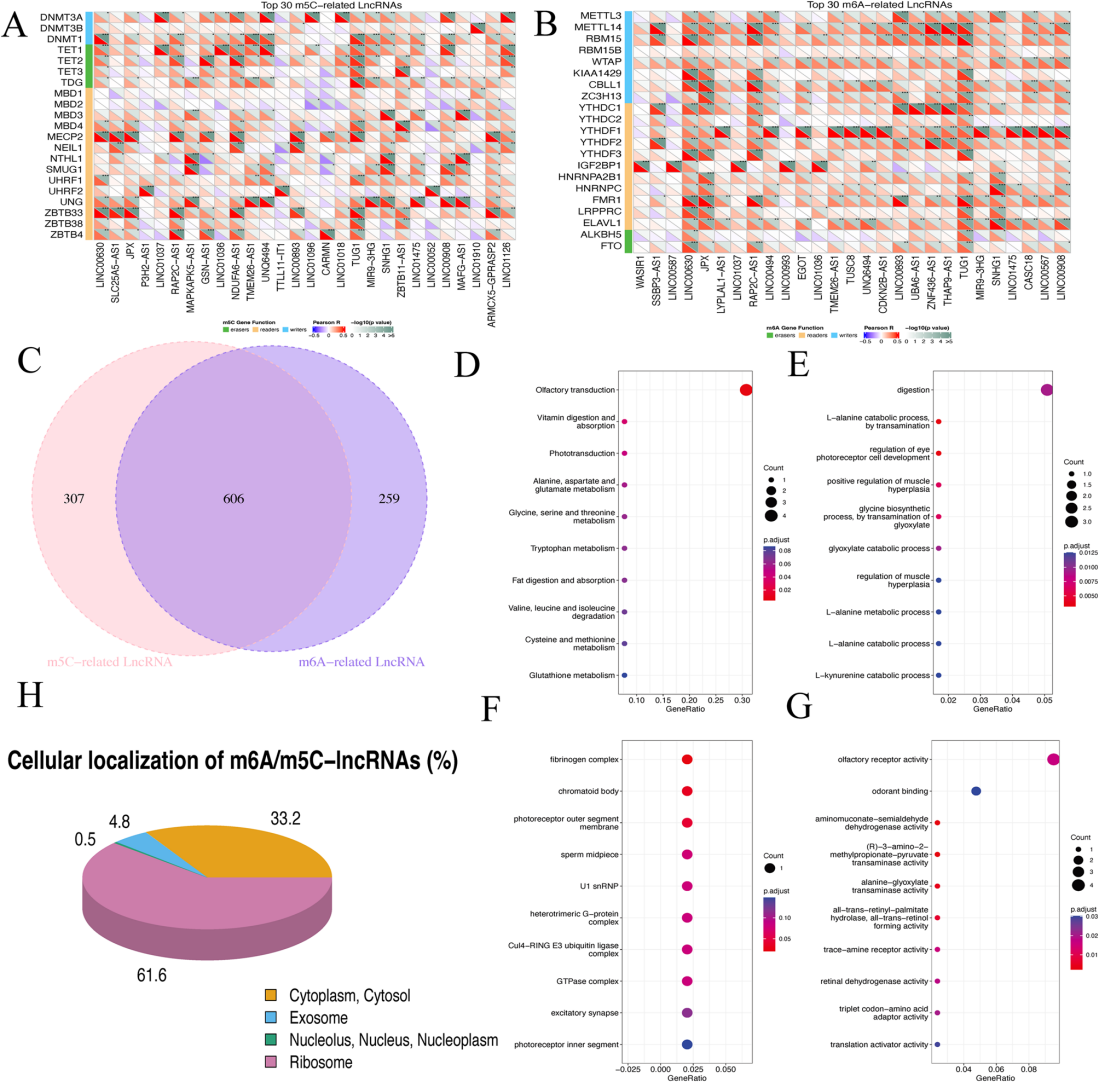
Identification of m6A/m5C-related lncRNAs in ESCC. (**A**,**B**) Top 30 m5C-related lncRNAs and top 30 m6A-related lncRNAs. (**C**) Overlapping lncRNAs between m5C- and m6A-related lncRNAs. (**D**) Functional enrichment analysis revealing KEGG pathways enriched by the lncRNA target genes. (**E**–**G**) Enrichment of GO biological process, cellular component, and molecular function. (**H**) Cellular localization of m6A/m5C-lncRNAs.

Using the defined criteria (|r|> 0.3 and P < 0.05), we identified a total of 2091 m5C gene-related lncRNAs and 2366 m6A gene-related lncRNAs within ESCC samples compared with normal samples. The top 30 lncRNAs related to m5C and m6A genes were listed in the correlation matrix (Fig. 1A,B). Based on the sequencing data in TCGA and GEO dataset, the lncRNA included in both two datasets were selected. Next, the overlapped lncRNAs between m5C and m6A gene-related lncRNAs were obtained as candidate molecules. Finally, we screened a total of 606 m6A/m5C-related lncRNAs for further analysis (Fig. 1C).

For these lncRNAs, we predicted their potential target genes by examining coding genes located 10 km upstream and downstream of each lncRNA, obtaining 758 target genes. We performed functional analysis, and found these m6A/m5C-lncRNA target genes were significantly enriched in the olfactory transduction pathway (Fig. 1D). Moreover, these genes demonstrated relevance to multiple biological process, cellular component, and molecular function, such as metabolism-related process, fibrinogen complex, chromatoid body, olfactory receptor activity and odorant binding (Fig. 1E–G).

In addition, we utilized the LncSLdb/lncATLAS database to predict the subcellular localization of lncRNAs. Our findings indicated that 61.55% of m6A/m5C-lncRNAs were localized in the ribosome, while 33.17% of lncRNAs were situated in the cytoplasm. Moreover, 4.79% of m6A/m5C-lncRNAs were found in exosomes, and 0.5% were identified in the nucleus (Fig. 1H).

### Molecular classification of ESCC case based on m6A/m5C-lncRNAs expression

**Figure 2:**
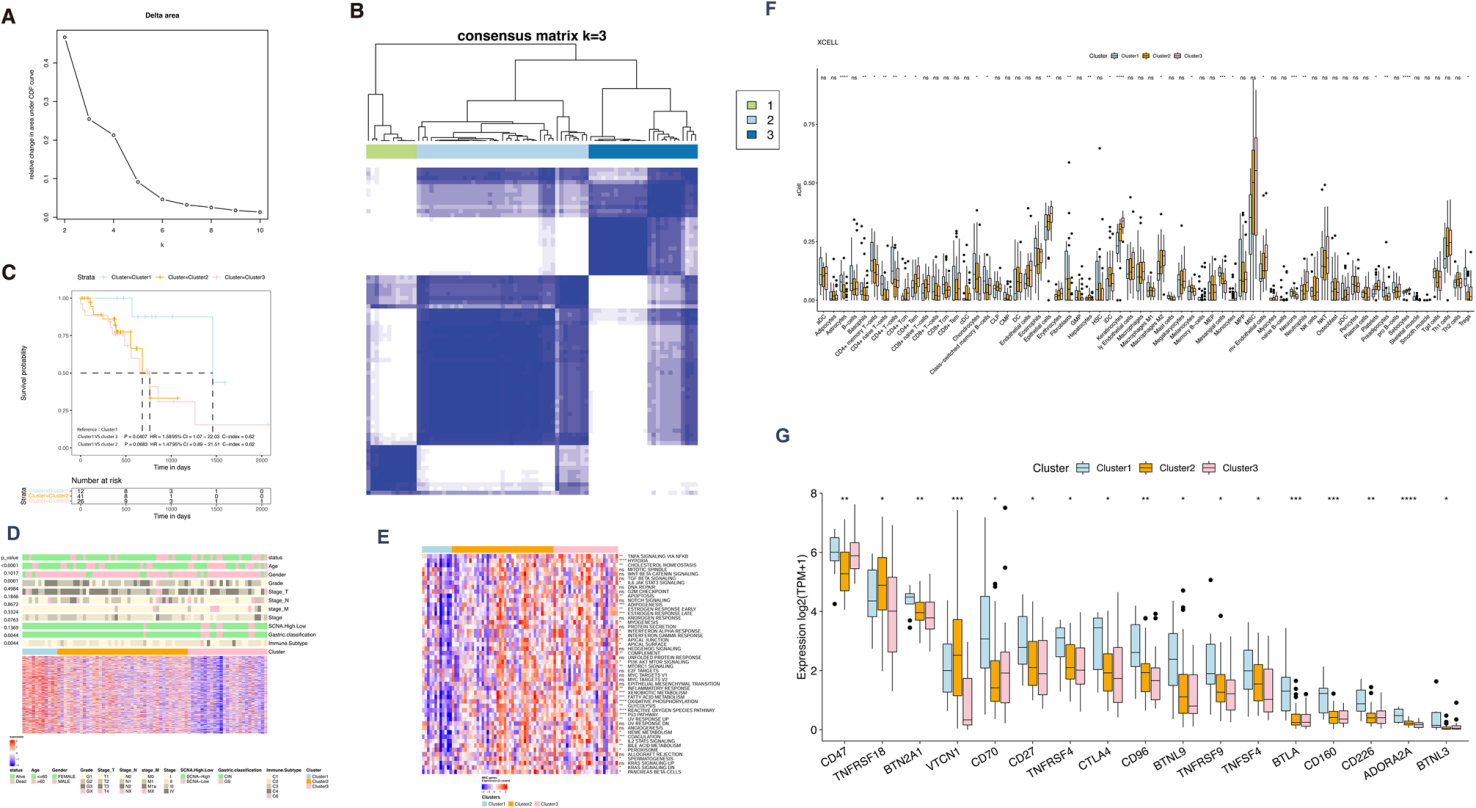
Identifying three clusters of ESCC cases by consensus clustering in the TCGA set. (**A**) Consensus clustering analysis and relative change in the area under the CDF curve. (**B**) Heat map of consensus matrix. (**C**) Prognosis analysis among the three clusters. (**D**) Heatmap of the clinical features among the three clusters in ESCC cases. (**E**) GSVA analysis displaying the key pathways enriched by 50 hallmark gene sets across three clusters. (**F**–**G**) Box plots illustrating differences in immunity landscape and immune checkpoint gene expression among three clusters.

Based on the expression differences of m6A/m5C-lncRNAs, we performed unsupervised classification of the ESCC cases in the TCGA dataset, ultimately identifying three distinct clusters (Fig. 2A,B). Survival analysis demonstrated significant variations in survival outcomes among these three clusters (Fig. 2C). Specifically, patients in cluster 1 exhibited superior survival outcomes compared to those in the other two clusters.

We further investigated the differences in various clinical features among the subtypes within the TCGA-ESCC dataset. The matrix results indicated that the m6A/m5C-lncRNAs expression in cluster 3 was relatively lower. In contrast, cluster 2 displayed moderate expression, while cluster 1 exhibited the highest expression levels of m6A/m5C-lncRNAs. Additionally, differences were observed in clinical pathological parameters, including age, grade, immune subtype, and gastric classification, across the three clusters (Fig. 2D).

Notably, significant differences were identified among the three clusters in the hallmark gene set. For instance, samples in cluster 1 displayed relatively lower GSVA enrichment scores across the 50 hallmark gene sets, while cluster 3 exhibited high scores. A total of 32 hallmark gene sets exhibited significant differences between the three clusters (Fig. 2E).

We analyzed immune landscape differences among the three distinct clusters using the xCell, CIBERSORT, and ssGSEA methods for the TCGA-ESCC samples. Our findings indicated that the infiltration ratio of CD4 + memory T cells, CD8 + T cells, and other T cell-related cell types was significantly lower in cluster 3, as evident in the xCELL results (Fig. 2F,G). Furthermore, a total of 17 immune checkpoint genes exhibited significant expression difference among the three clusters. Notably, most of these genes were markedly upregulated in cluster 1 compared to cluster 3 (Fig. 2G).

### Construction of m6A/m5C-lncRNA-based prognostic signature

**Figure 3:**
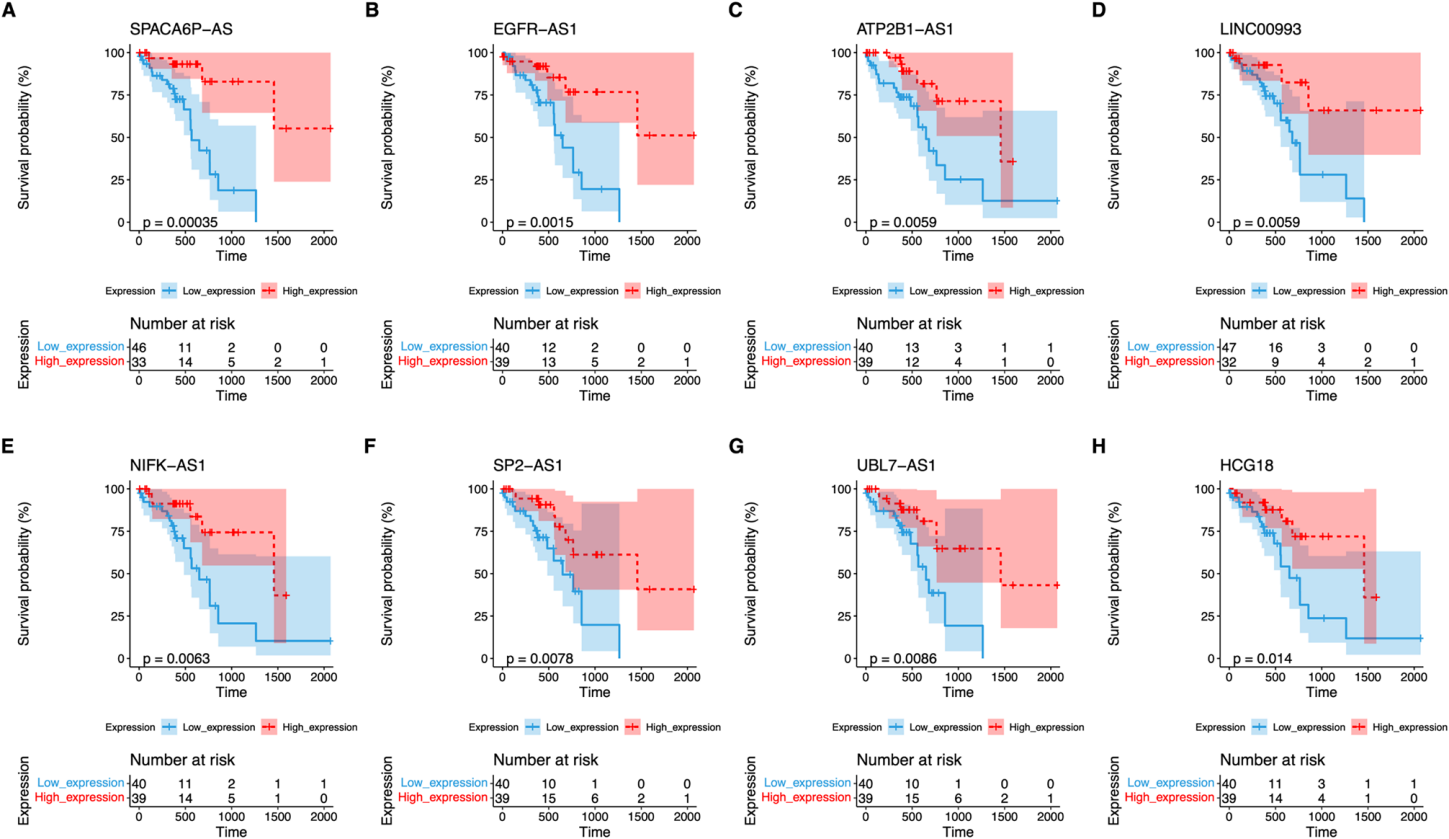
Survival analysis results of prognostic lncRNAs between high- and low- expression groups of ESCC samples.

To identify the prognostic lncRNAs, we conducted a cox regression analysis based on the TCGA-ESCC samples. There were 17 m6A/m5C-lncRNAs with significant prognostic differences (Table 2). We classified the ESCC cases into higher and lower expression groups using the median gene expression value as the criterion. Survival analysis results demonstrated significant prognostic differences among 8 of these lncRNAs between the high- and low-expression groups (Fig. 3).

**Table 2:** Univariate cox regression analysis showing the m6A/m5C-related prognostic lncRNAs.

Gene	coef	p.value	Hazard_ratio	Lower_.95	Upper_.95	Log rank pvalue	Wald pvalue	Likelihood_pvalue	HR
LINC00898	− 0.291339	0.00167241	0.74726232	0.62311853	0.89613926	0.00125613	0.00167241	0.00292077	0.75 (0.62 − 0.9)
LINC00847	1.1596129	0.00240528	3.18869869	1.50795139	6.74278988	0.00221678	0.00240528	0.00105983	3.19 (1.51 − 6.74)
LINC01415	− 0.3823088	0.00261062	0.68228435	0.53193629	0.87512723	0.00308498	0.00261062	0.00876798	0.68 (0.53 − 0.88)
LINC00993	− 0.2139095	0.01601545	0.80742144	0.67842728	0.96094218	0.01004049	0.01601545	0.00552336	0.81 (0.68 − 0.96)
EGFR-AS1	− 0.3346057	0.00933494	0.71562018	0.55605549	0.92097327	0.01120361	0.00933494	0.01302588	0.72 (0.56 − 0.92)
LINC01602	− 0.201695	0.01547201	0.81734415	0.69421638	0.96231013	0.01305807	0.01547201	0.00907101	0.82 (0.69 − 0.96)
XIST	− 0.1318115	0.02867726	0.87650617	0.77888553	0.98636197	0.02146775	0.02867726	0.01243166	0.88 (0.78 − 0.99)
MIF − AS1	− 0.4483249	0.02921312	0.63869717	0.42686483	0.95565162	0.02690006	0.02921312	0.03236641	0.64 (0.43 − 0.96)
TTTY15	0.16123551	0.03157228	1.17496165	1.01433849	1.3610199	0.02879093	0.03157228	0.02035923	1.17 (1.01 − 1.36)
LINC00208	− 0.6352842	0.0648618	0.52978488	0.26989787	1.03991935	0.03195375	0.0648618	0.009065	0.53 (0.27 − 1.04)
LINC01393	0.36344936	0.02849482	1.43828203	1.03897716	1.99104973	0.03489665	0.02849482	0.01637473	1.44 (1.04 − 1.99)
LINC01037	− 0.1297853	0.04422671	0.87828402	0.77397328	0.99665304	0.03599402	0.04422671	0.02655686	0.88 (0.77 − 1)
LINC00942	0.12213836	0.04113541	1.12991042	1.00492827	1.27043652	0.03952849	0.04113541	0.04785422	1.13 (1 − 1.27)
LINC00638	− 0.3722211	0.0409009	0.68920183	0.48236903	0.98472153	0.04014923	0.0409009	0.04277191	0.69 (0.48 − 0.98)
LINC00415	− 0.1628923	0.05239957	0.8496827	0.7207415	1.00169158	0.04029506	0.05239957	0.02560894	0.85 (0.72 − 1)
LINC01310	− 0.3170921	0.05800911	0.72826368	0.52469096	1.01081977	0.04475305	0.05800911	0.02244852	0.73 (0.52 − 1.01)
LINC01036	− 0.1226793	0.05475261	0.88454726	0.78047092	1.00250225	0.0485204	0.05475261	0.04237056	0.88 (0.78 − 1)

**Figure 4:**
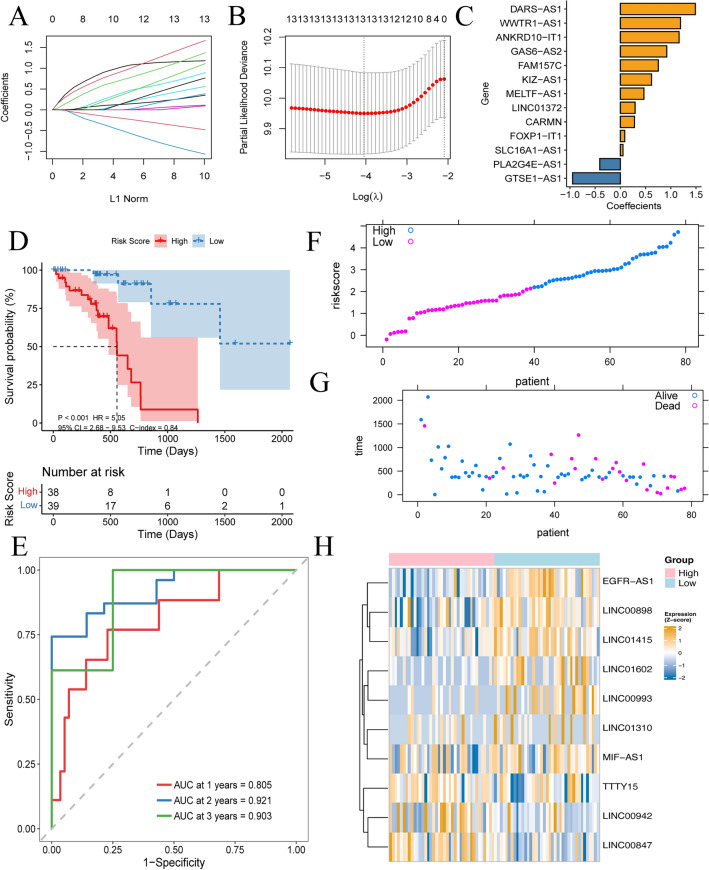
Construction of lncRNAs signature-based RiskScroe system for prognosis prediction. (**A**,**B**) Construction of the RiskScore model using the lasso method. (**C**) Lasso coefficients of the 10 prognostic genes. (**D**) Survival analysis of ESCC patients in high- and low- RiskScore cohorts. (**E**) ROC curve of the RiskScore at 1-, 2- and 3-year follow-up. (**F**–**H**) Distribution of RiskScore, status, and expression of m6A/m5C-lncRNAs in the TCGA.

According to lasso-cox analysis, we finally obtained 10 prognostic lncRNAs, including LINC00847, LINC00942, TTTY15, LINC01602, LINC01310, LINC00898, EGFR-AS1, MIF-AS1, LINC00993, and LINC01415. A ten lncRNAs-based RiskScore model was generated for survival prediction (Fig. 4A–C).

In the training set, we observed that patients in the high-RiskScore subtype had a worse prognosis than those in the low-RiskScore group (Fig. 4D,F–H). Furthermore, ROC curve analysis indicated that this model exhibited promising predictive ability for survival in TCGA-ESCC samples (AUC value at 1-, 3-, and 5- years were respectively 0.805, 0.921 and 0.903; Fig. 4E).

### Validation of the RiskScore model

**Figure 5:**
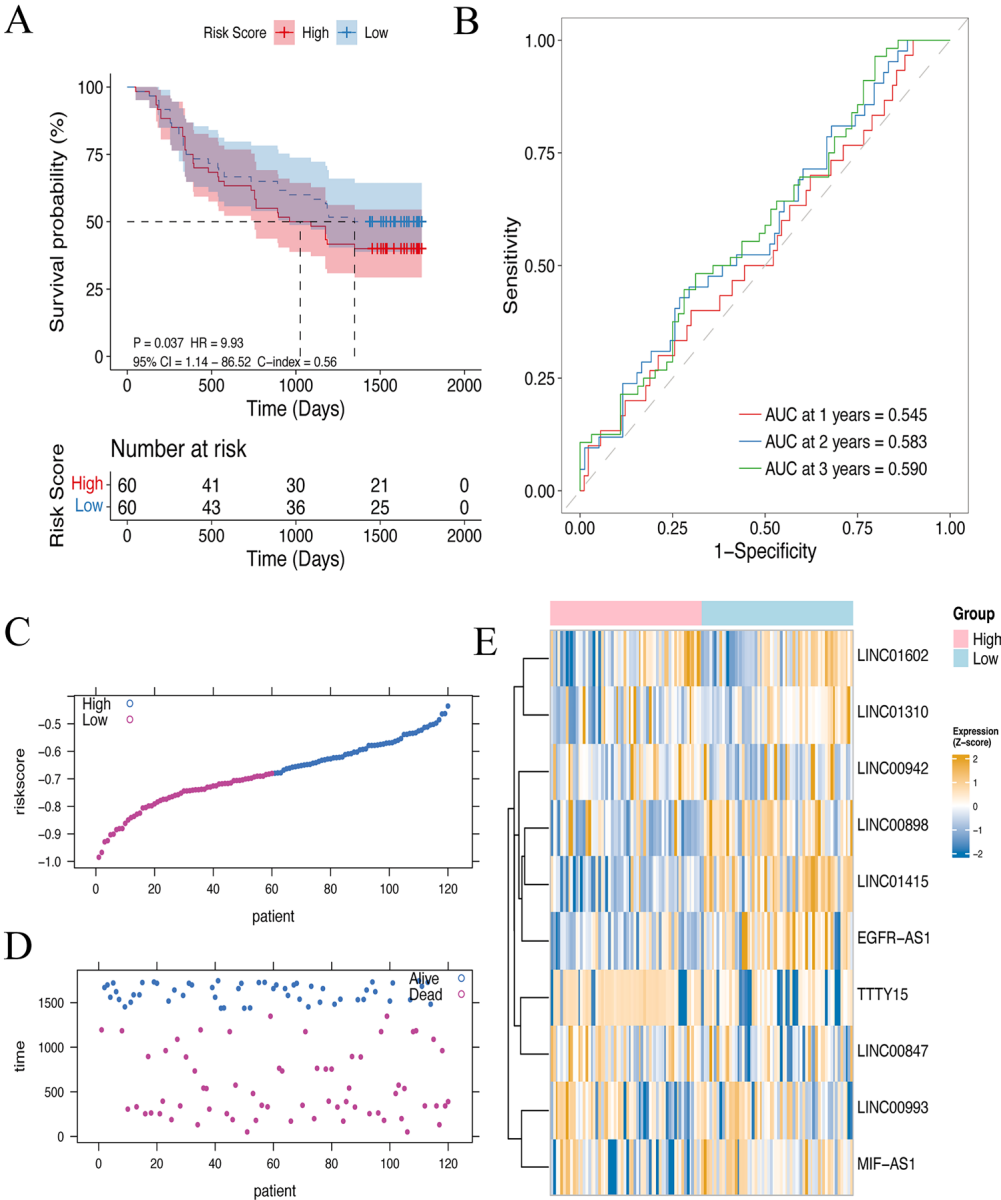
Prognostic value of RiskScore models in an external validation set. (**A**) Survival analysis of two subgroups via the GSE53622 dataset. (**B**) ROC curve analysis of the RiskScore model in the validation set. (**C**–**E**) Distribution of RiskScore, status, and the expression level of lncRNAs in the GSE53622 dataset.

The predictive ability was also evaluated in the validation set GSE53622. There was a significant prognostic difference between the two subgroups (Fig. 5A,C–E). ROC curve confirmed the superior predictive power of this RiskScore model in the validation sets (AUC values at 1-, 2-, and 3- years were respectively 0.545, 0.583, 0.590; Fig. 5B).

**Figure 6:**
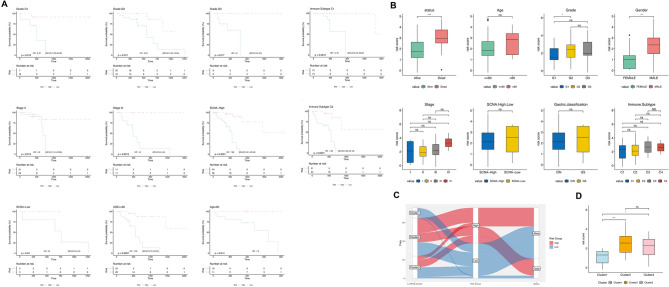
Prognostic efficiency prediction of m6A/m5C-lncRNA signatures. (**A**) Predictive value of RiskScore in clinicopathological subgroups. (**B**) RiskScore assessments across different clinicopathological groups, including survival status, age, grade, gender, stage, somatic copy number alterations (SCNA) levels, and gastric classification. (**C**) Sankey diagram visualized the correlation ship of clusters, RiskScore group and survival status. (**D**) Risk score assessments between different cluster cohorts.

Next, the prognostic efficiency of the RiskScore system in various clinical feature subgroups was confirmed on the TCGA-ESCC samples. The results demonstrated significant survival differences between the high- and low- RiskScore groups in different clinical characteristics, age, grade, stage, immune subtype, and SCNA level (Fig. 6A).

Furthermore, ESCC patients in the TCGA dataset could be classified into different subgroups based on clinical features, such as stage, grade, and age. RiskScore assessments were performed between different clinical feature groups and the previously identified three clusters. Notably, patients in the alive and female subgroups presented a low RiskScore, indicating a significant correlation between RiskScore and survival status and gender. Moreover, a significant difference in RiskScore was observed between the G1 and G2 grade subgroup (*P* < 0.05, Fig. 6B). Patients in cluster 1 displayed the lowest RiskScore than the other two clusters (*P* < 0.05, Fig. 6D). Sankey diagram showed the consistency between cluster and RiskScore for survival status prediction, which exhibited a consistent result to the RiskScore assessments (Fig. 6C,D).

### Identification of RiskScore as an independent prognostic factor

**Figure 7:**
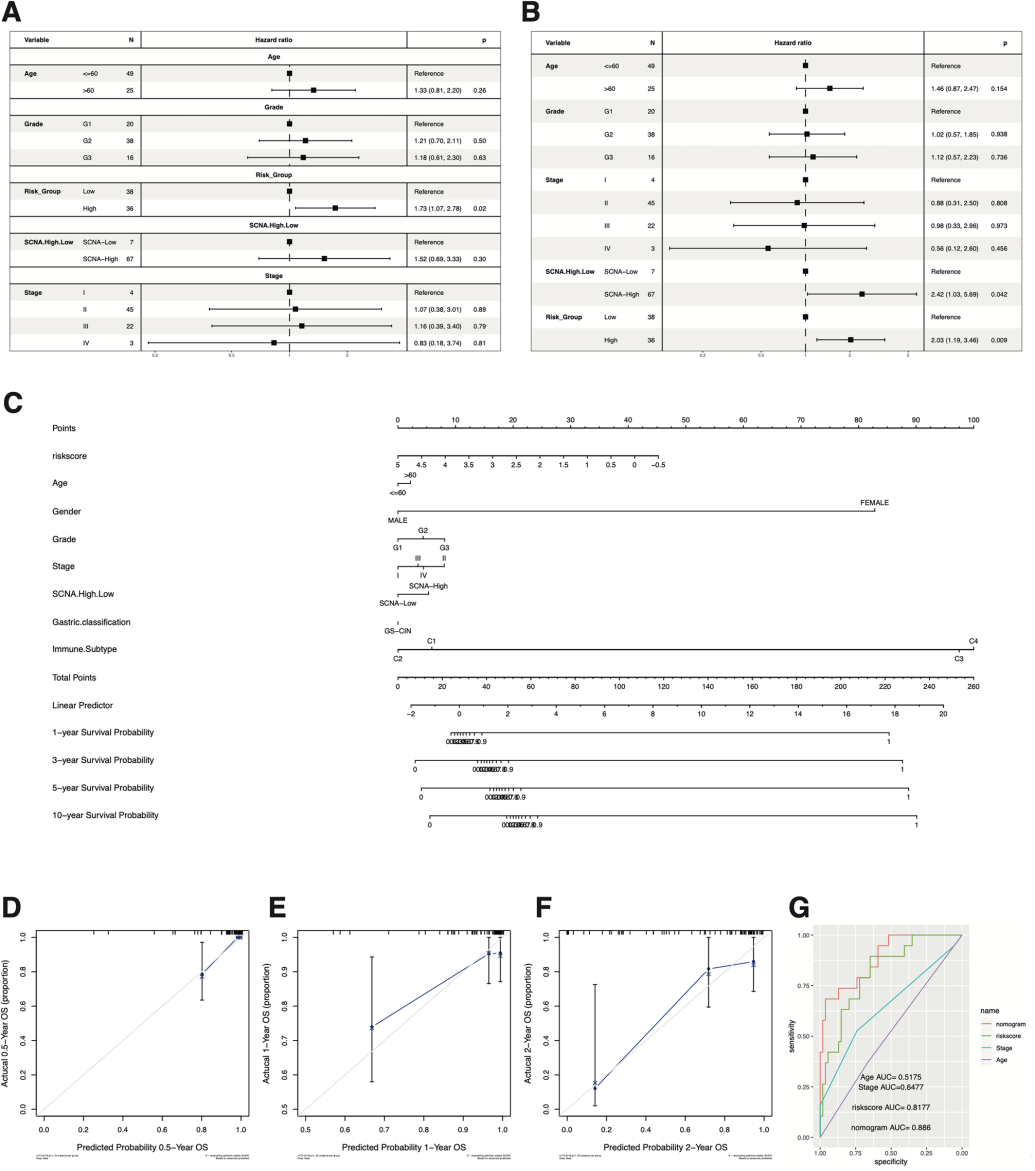
Cox regression analysis and nomogram model construction for m6A/m5C-lncRNAs prognostic signature. (**A**,**B**) Cox regression analysis for the survival time in TCGA cohorts. (**C**) The nomogram model predicting survival outcomes of ESCC. (**D**–**G**) ROC and calibration curves for predicting the survival time in the TCGA cohort.

Utilizing survival information from the TCGA-ESCC dataset, we conducted a cox regression analysis, and the results revealed that RiskScore emerged as an independent prognostic factor for survival prediction (Fig. 7A,B; *P* < 0.05, *P* < 0.01).

We constructed a nomogram model that integrated clinical characteristics and RiskScore for predicting survival in the TCGA (Fig. 7C). To gauge the predictive accuracy, we employed ROC curves, revealing AUC values of 0.518 and 0.648 for age and stage, respectively (Fig. 7G). The nomogram prediction and RiskScore possessed higher AUC values (0.886, 0.818) than other clinically relevant prognostic factors in the TCGA cohort. It is worth noting that due to limited data, we refrain from drawing generalized conclusions beyond 3 years. The calibration curves demonstrated consistency with the standard curve in predictive outcome at 0.5, 1, and 2 years (Fig. 7D–F). These findings revealed nomogram model exhibited reliable prognostic predictive power compared with other clinical characteristics in TCGA cohorts.

### Association of RiskScore and several highly trustworthy indices

Cancer progression encompasses the transition towards a dedifferentiated oncogenic phenotype and the acquisition of stem cell-like features. A previous study employed the innovative logistic regression OCLR algorithm to introduce two stemness indices for quantifying tumor microenvironment stemness^22^: mRNA expression and the methylated DNA-based stemness index (mRNAsi and mDNAsi). These indices reflect the gene expression and epigenetic characteristics of stem cells. Epigenetic regulation-based mRNAsi and mDNAsi (EREG-mRNAsi and EREG-mDNAsi) were generated via reconstructing the gene regulatory network from methylation and transcriptome data^22^. Additionally, single nucleotide variants (SNVs) represent the most prevalent genomic mutations in tumor cells, leading to the production of variant peptides distinct from wild-type proteins, which are subsequently presented by MHC-I^23^.

**Figure 8:**
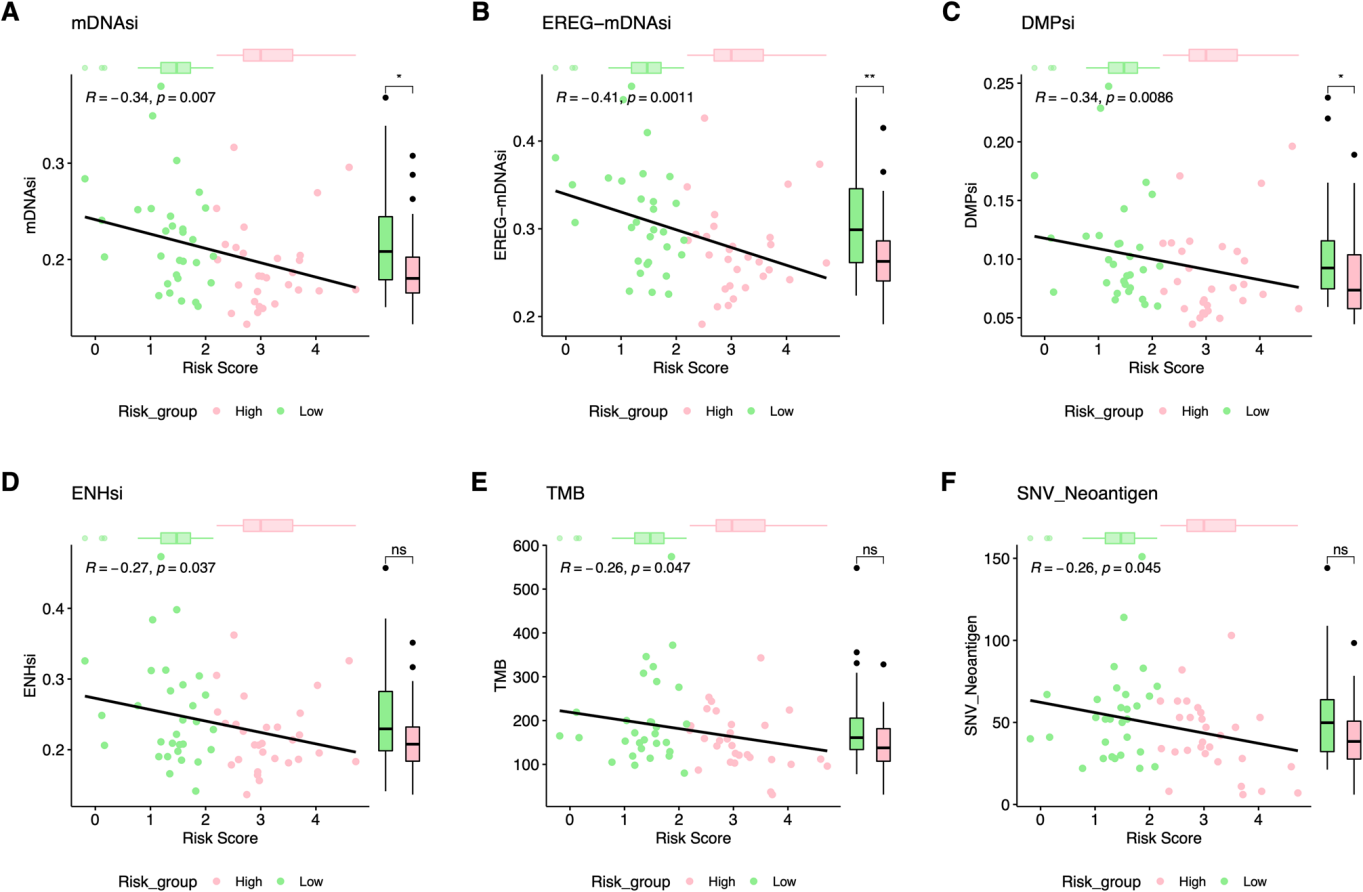
Correlation analysis between RiskScore and several clinical trustworthy indices. *P < 0.05; **P < 0.01; ***P < 0.001.

In this context, we explored the correlation between RiskScore and several highly reputable indices within the TCGA-ESCC cohort. These indices encompassed stemness indices (mRNAsi, mDNAsi, EREG-mDNAsi, DMPsi), tumor mutational burden (TMB), stromal score, tumor purity, immune score, and SNV-neoantigen. Our findings unveiled a negative correlation between the m6A/m5C-lncRNA signature and mDNAsi, EREG-mDNAsi, DMPsi, ENHsi, TMB, and SNV-neoantigen. Further analysis revealed significant statistical differences between the two groups in mDNAsi, DMPsi, and EREG-mDNAsi. Particularly noteworthy was that ESCCs in the high-RiskScore groups exhibited lower mRNAsi, DMPsi, and EREG-mDNAsi compared to the low-RiskScore group (Fig. 8, *P* < 0.05).

### Correlation between lncRNA signature and immune cell proportion

**Figure 9:**
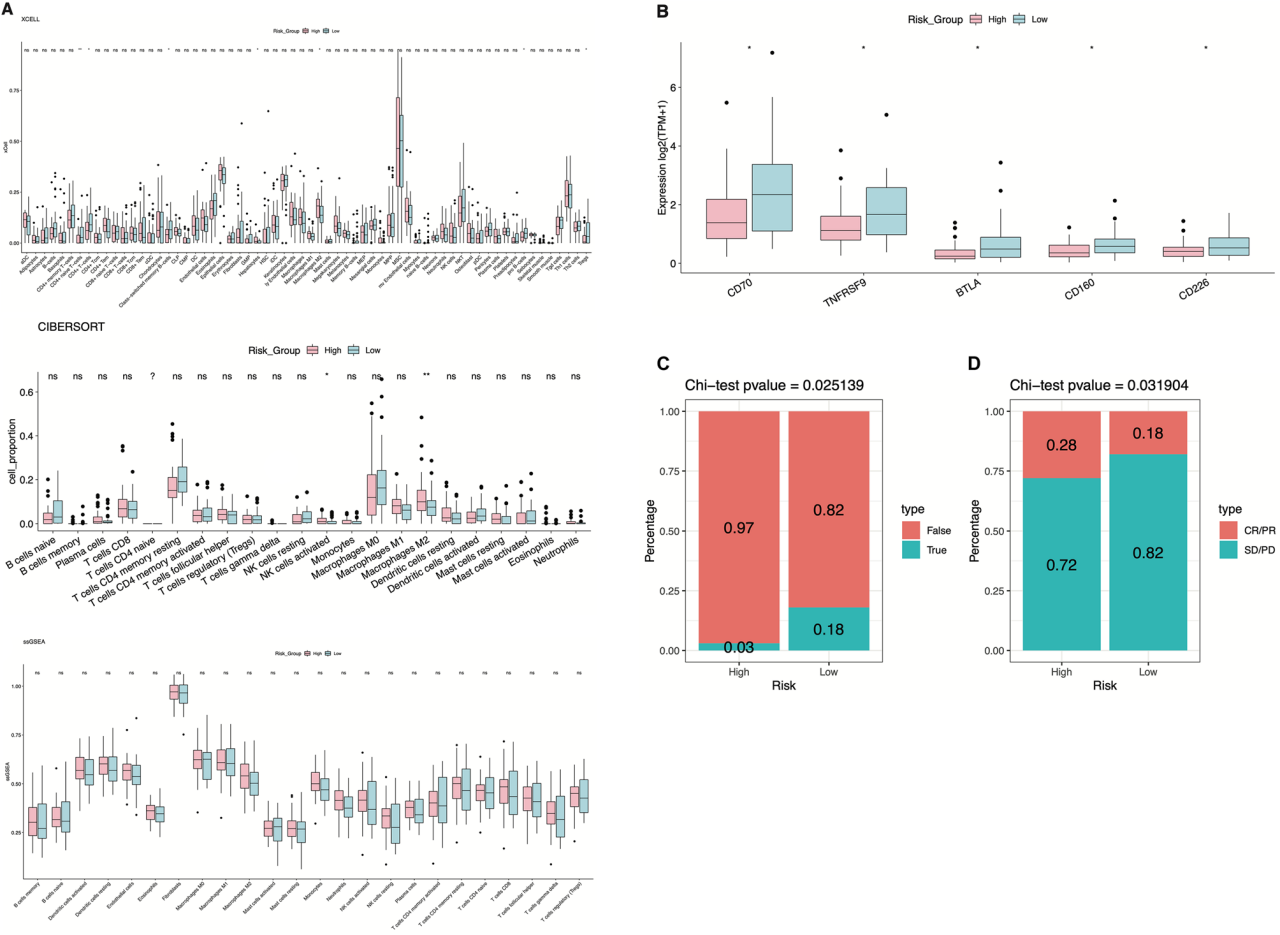
Association of RiskScore, infiltrated immune cell and immunotherapy response via TCGA-ESCC datasets. (**A**,**B**) Differences in immune cell infiltration and immune checkpoint gene expression between two subgroups. *P < 0.05; **P < 0.01; ***P < 0.001. (**C**) Differences in immunotherapy efficacy between two risk subgroups in TCGA-ESCC cohorts. (**D**) Immunotherapy response between high and low-RiskScore groups in the IMvigor210 datasets.

We evaluated the immune cell ratio in the TCGA-ESCC dataset between the two risk groups. Based on xCell results, we observed a significantly increased ratio of CD4 + T cells in the low-RiskScore group compared to the high-RiskScore group (Fig. 9A, P < 0.05). Conversely, the cibersort results indicated a decreased proportion of activated NK cells and macrophage M2 in the low-RiskScore group (Fig. 9A, P < 0.05). However, ssGSEA analysis revealed no statistical difference in infiltrated immune cell levels between the high and low-RiskScore groups (Fig. 9A, P < 0.05).

Additionally, we analyzed the expression of immune checkpoint genes between the high and low RiskScore groups. Our findings revealed that five immune checkpoint genes exhibited significantly abnormal expression between the two groups, with their expression levels in the low-risk group being notably higher than those in the high-RiskScore group (Fig. 9B).

Moreover, we assessed the predictive value of RiskScore in immunotherapy efficiency using the TIDE method on TCGA-ESCC data. The results indicated that the low-RiskScore group was more likely to respond to immunotherapy compared with high- RiskScore patients (*P* = 0.025, Fig. 9C). Similarly, we analyzed the proportions of patients who responded to immunotherapy in the high and low-RiskScore groups using the IMvigor210 dataset. Our findings revealed a significant difference in the percentage of drug response patients between the two subgroups (*P* = 0.032, Fig. 9D), underscoring the reliable predictive performance of the RiskScore model in immunotherapy response.

## Discussion

In the treatment of patients with cancer, immunotherapy has emerged as a pivotal therapeutic avenue in the management of gastrointestinal cancers due to its potential to yield rapid and substantial therapeutic benefits for afflicted patients. However, it remains a huge challenge to screen the dominant populations likely to mount a robust response to immune checkpoint inhibitors (ICIs). Here, our findings unveil a strong correlation between low-RiskScores and favorable responses to immunotherapy, signifying the potential use of this system in patient stratification for enhanced therapeutic outcomes. LncRNA represents a diverse category of RNA molecules with intricate roles in regulating gene expression by engaging gene regulatory proteins and microRNAs. Similar to protein-cording RNAs, lncRNAs also undergo RNA methylation that contributes to their regulatory functions in the context of tumorigenesis.

In this study, we analyzed the transcriptomic profiles from public databases and uncovered 606 m6A/m5C-related lncRNAs in ESCC. By consensus clustering analysis, we classified these ESCC samples into three clusters based on lncRNA expression profiles and found that cluster1 was associated with a significant better prognosis than cluster 2 and cluster 3. The survival difference reason was investigated by comparing the immune landscape. As we expected, patients in cluster 1 displayed higher immune cell proportions in CD4 memory T cells and CD8 T cells. Furthermore, most of the immune checkpoint genes were notably increased in cluster 1 than the other two subgroups, indicating that immunity plays a major role in ESCC prognosis.

We employ the lasso method and survival analysis to identify ten m6A/m5C-related lncRNAs associated with ESCC prognosis, including LINC00847, TTTY15, and LINC00942. Notably, LINC00847 has been extensively studied in various cancer types. It emerges as a key player in laryngeal squamous cell carcinoma, and its overexpression has been linked to enhanced cell proliferation and cell cycle progression. Additionally, elevated LINC00847 levels are correlated with lymph node metastasis and poor differentiation, suggesting its potential as a prognostic biomarker. Our results further corroborate the significance of LINC00847 in ESCC survival, consistent with previous research. Mechanistically, LINC00847 is induced by the transcription factor E2F, exerting its influence on cancer progression through the modulation of downstream targets, including miR-147a^24^.

In addition, we developed a ten m6A/m5C lncRNAs-based RiskScore model, which had a promising ability in survival prediction for both TCGA cohorts and independent validation datasets. Patients stratified into high and low-RiskScore subgroups manifest conspicuous distinctions in key clinical characteristics, encompassing survival status, gender, tumor stage, and grade. Additionally, the RiskScore model unveiled significant associations with immune cell infiltration, notably affecting CD4 + T cells and Tregs proportion. To further improve its clinical utility, we constructed a comprehensive nomogram model incorporating clinical variables and RiskScore. This model performed excellent in predicting the survival probability of ESCC.

Moreover, emerging evidence highlights that tumor-infiltrating immune cells were correlated with prognosis and immunity. The composition changes and functional activation of immune cell populations within the TME exert a profound impact on patient survival and their responsiveness to immunotherap^25^. For instance, augmented infiltration of CD4 + lymphocytes has consistently demonstrated a significant association with prolonged survival in ESCC^26^. In this study, we also characterized increased CD4 + T cell infiltration in low-RiskScore group, which displays a better prognosis. This observation aligns seamlessly with prior research, further reinforcing the notion that increased CD4 + T cell infiltration might be indicative of a more favorable prognosis.

Interestingly, we found the immune checkpoint genes were upregulated in the low-RiskScore group. Among these genes, CD226 deficiency restrains CD8 + T cell function, consequently curtailing the efficacy of cancer immunotherapy^27^. Aberrant expression of CD70 was linked to tumor progression and immunosuppression in the tumor microenvironment, and it can facilitate immune evasion through interacting with receptor CD27^28^. TNFRSF9 is an activation marker for tumor-infiltrating Tregs, and inhibition of TNFRSF9 boosts anti-cancer treatments via reducing the immune suppressive function of Tregs^29^. These compelling findings collectively imply a potential mechanism whereby m6A or m5C methylation on lncRNAs may exert regulatory control over immune checkpoint gene expression. Such regulation contributes to a positive response to immunotherapy in the context of ESCC.

More and more studies have confirmed that lncRNA has a close relationship with the immune microenvironment. Studies have confirmed that most m6A regulators play an important role in the immune microenvironment by activating the PI3K-AKT pathway and enriching infiltrating immune cells^30^. m6A phenotype-related genes were obtained by identifying the DEGs associated with m6A phenotype and overlapping them. m6A phenotype-related genes involved in immune regulation were screened out, and the enriched biological processes were significantly related to antigenic process, Fc signal transduction, immune cell proliferation regulation and cytokine-mediated pathways. Meanwhile, Zhang et al.^31^ searched for highly correlated gene clusters associated with immune cells through the algorithm WGCNA. The feature genes of related gene clusters were clustered, and the gene-lncRNA modules related to immune cell score and clinical features were finally determined. An additional integrative analysis of the single-cell RNA-seq dataset has unveiled a compelling close association between LINC00847 and prognosis in lung adenocarcinoma. LINC00847 positively correlates with the infiltration of various immune cell types, and its overexpression significantly down-regulates PDL1 expression in the in-vitro assay, thus casting it as a prospective candidate in tumor immunotherapy^32^. Furthermore, the male Y chromosome-linked lncRNAs TTTY15 assume a crucial role in carcinogenesis across diverse cancer types. Acting as an RNA sponge, TTTY15 engages several miRNAs to promote cancer progression, exemplified by its interaction with miR-29-3p in colorectal cancer^33^, miR-98-5p^34^ and miRNA let-7a-5p^35^ in gastric cancer. In prostate cancer, TTTY15 exhibits prominent upregulation in most tumor samples, exerting a pro-carcinogenic influence by sponging miRNA let-7, subsequently elevating CDK6 and FN1 expression^36^. Interestingly, TTTY15 assumes a contrasting suppressive role in NSCLC, wherein its overexpression inhibited cancer proliferation and metastasis through the modulation of TBX4^37^.

Moreover, epigenetic modification of lncRNAs in regulating tumorigenesis and development has been reported. In breast cancer, it has been documented that LINC00942 directly engages methyltransferase METTL14, thereby facilitating METTL14-mediated RNA methylation processes downstream^38^. Moreover, LINC00942 has been implicated in promoting chemoresistance in gastric cancer by impeding the degradation of oncoprotein MSI2. This, in turn, enhances the stability of c‐myc mRNA, a phenomenon reliant on m6A modification^39^. Another lncRNA of interest, EGFR-AS1, arising from the reverse strand of lncRNA EGFR, has garnered attention for its overexpression in diverse cancer types. Elevated levels of EGFR-AS1 have been closely associated with unfavorable clinical features, encompassing pathological stage, lymph node metastasis, and overall survival^40^. Functionally, EGFR-AS1 serves as an oncogene, fostering cell proliferation, chemotherapy resistance, invasion, and stemness through intricate interactions with downstream miRNAs and signaling pathways. EGFR-AS1 notably stabilizes EGFR mRNA, consequently activating the PI3K/AKT pathway to promote proliferation and metastasis in renal cancer cells^41^.

Furthermore, EGFR activation has been linked to the up-regulation of PDL1 via the p-ERK1/2/p-c-Jun pathway, thereby inducing immune evasion in EGFR-driven cancer^42^. In squamous cell carcinoma, EGFR-AS1 emerges as a mediator of EGFR addiction, influencing treatment responses. Notably, EGFR-AS1 knockdown has been shown to reverse resistance to tyrosine kinase inhibitors^43^. In ESCC, EGFR-AS1 has been implicated in up-regulating ROCK1 expression by sponging miR-145, thus promoting cancer cell invasion and migration^44^. Additionally, lncMIF‐AS1 has demonstrated its significance in positively regulating NDUFA4 expression in gastric cancer cells. This regulation is achieved through the sequestration of miR‐212‐5p, which attenuates NDUFA4 mRNA degradation. Upregulation of NDUFA4 activates the oxidative phosphorylation pathway, ultimately promoting proliferation and inhibiting apoptosis in gastric cancer cells^45^. It is noteworthy that the biological roles of the remaining five lncRNAs remain unclear, thus providing new directions for future research endeavors. Collectively, these findings illuminate the indispensable role of m6A/m5C-lncRNAs in cancer development. Moreover, they posit these lncRNAs as promising candidates for novel prognostic biomarkers, holding potential implications for the prognosis of patients afflicted with ESCC.

In summary, our finding demonstrated a ten m6A/m5C-lncRNA signature implicated in ESCC progression and established a lncRNA signature-based RiskScore model for prognosis prediction. Moreover, we assessed the immune landscape and immune checkpoint gene expression for low-RiskScore patients, which might contribute to a beneficial therapeutic response from ICI. The RiskScore system might be a useful tool to determine the m6A/m5C-lncRNA signature application in clinical practice, thus promoting treatment decisions for selecting the patient subgroup that more cline benefits from ICI therapy.

Certain limitations should be mentioned in our context. Firstly, since the data were analyzed through TCGA and GEO databases, there is still a lack of verification of wet laboratory biochemical experiments. Second, our results of m6A/m5C-associated lncRNAs signature were not validated in a separate patient cohort, and the survival predictive value of the RiskScore model requires more external datasets validation for clinical application. Finally, the potential molecular mechanism of these m6A/m5C-related lncRNAs remained unclear in ESCC, and we plan to conduct in vitro or in vivo biological experiments to verify our results in future studies.

## Conclusion

In this study, ten m6A/ m5C-lncRNA signals were identified to be associated with ESCC progression. Molecular subgroup analysis based on m5C- and m6A-associated lncRNAs was performed on ESCC samples, and m5C- and m6A-associated lncRNAs related molecular subtypes were obtained, which confirmed significant prognostic differences between subtypes. By establishing the RiskScore model of lncRNA associated with m5C and m6A, we found that there were significant differences between the high and low risk groups regardless of the tumor micro-environment landscape or immune-related genes. At the same time, to further improve the clinical utility of the model, a comprehensive column line model including clinical variables and risk scores was constructed. The model performs well in predicting the survival probability of ESCC. Therefore, we believe that the RiskScore model has a good prognostic power. In addition, RiskScore model also has generalized value in chemotherapy prediction. The results are remarkable, and the analysis methods are solid, diverse and comprehensive. However, this risk score model is built on a public database and needs to be validated in a clinical setting. Bias from some unmeasured clinical variables may have weakened the statistical validity of our study. In the future, we will verify our results in a clinical study.

## Data Availability

The datasets used and/or analysed during the current study available from the corresponding author on request.

## Abbreviations

AUC: Area under the curve
CPS: Combined positive score
DMPsi: Differentially methylated probes stemness index
EREG-mDNAsi: Epigenetically regulated DNA methylation-based index
ESCC: Esophageal squamous cell carcinoma
GEO: Gene expression omnibus
ICI: Immune checkpoint inhibitor
lncRNA: Long non-coding RNAs
m5C: 5-Methylcytosine
m6A: N6-methyladenosine
mDNAsi: DNA methylation-based stemness index
mRNAsi: RNA methylation-based stemness index
OCLR: One‐class logistic regression
ROC: Receiver operating characteristic
SNV: Single-nucleotide variants
TPS: Tumor proportion score
